# Draft Genome Sequence of *Nocardia seriolae* ZJ0503, a Fish Pathogen Isolated from *Trachinotus ovatus* in China

**DOI:** 10.1128/genomeA.01223-14

**Published:** 2015-01-02

**Authors:** Liqun Xia, Jia Cai, Bei Wang, Yucong Huang, Jichang Jian, Yishan Lu

**Affiliations:** a Fisheries College, Guangdong Ocean University, Zhanjiang, China; b Guangdong Provincial Key Laboratory of Pathogenic Biology and Epidemiology for Aquatic Economic Animals, Zhanjiang, China; c Key Laboratory of Diseases Controlling for Aquatic Economic Animals of Guangdong Higher Education Institutions, Zhanjiang, China

## Abstract

*Nocardia seriolae* is a pathogen that causes nocardiosis in marine and freshwater fish. Here, we report the draft genome sequence of *N. seriolae* strain ZJ0503, which was isolated from *Trachinotus ovatus* in Guangdong, China.

## GENOME ANNOUNCEMENT

*Nocardia seriolae* is an important pathogen frequently associated with nodules and skin ulcers in fish (1). Over the last few years, *N. seriolae* has been an emerging pathogen that has caused increasing damage in both freshwater and marine aquaculture systems in Asia (2, 3). Until now, little was known about the pathogenic mechanism of *N. seriolae*. The *N. seriolae* strain ZJ0503, which was isolated from diseased *Trachinotus ovatus* in Guangdong Province, China, was chosen for sequencing. The draft genome sequence of *N. seriolae* strain ZJ0503 was determined using Illumina MiSeq at Novogene Bioinformatics Technology Co., Ltd. (Beijing, China). Draft assemblies were based on 1,008-Mb reads. All reads provided about 130-fold coverage of the genome. The MiSeq paired-end reads were assembled into 319 contigs in 315 scaffolds with the SOAPdenovo program. Gaps were closed by all the paired-end reads. Putative open reading frames with more than 30 amino acid residues were predicted using GeneMarkS (4). Interspersed repeat sequences (IRSs) and tandem repeat sequences (TRSs) were predicted using RepeatMasker (5). rRNAs, tRNAs, and sRNA were identified using RNAmmer (6), tRNAscan (7), and Rfam (8), respectively. The scaffolds were searched against the GO (Gene Ontology), KEGG (Kyoto Encyclopedia of Genes and Genomes), COG (Clusters of Orthologous Groups), NR (Non-Redundant Protein), Swiss-Prot, and TrEMBL databases to annotate the gene descriptions. The genome of strain ZJ0503 contains 7,708,091 nucleotides and has a G+C content of 68.25%. There are 7,426 coding sequences (CDSs) that account for 86.32% of the genome. IRSs and TRSs account for 0.055% and 0.465% of the genome, respectively. 62 tRNAs,1 rRNAs, and 105 sRNAs were predicted.

Just like other *Nocardia* species (9), some drug-resistant genes, such as rpoB2, FAR-1, β-lactamase, monooxygenase, FolP, and efflux pump, are also found in the genome of ZJ0503, which may explain why *N. seriolae* is naturally resistant to many antibiotics. CDSs for a few potential virulence-associated factors of *N. seriolae*, such as mammalian cell entry (mce) family protein, ATP-binding cassette (ABC) transporters, capsular polysaccharides (CPS), Myosin-cross-reactive antigen (MCRA), fibronectin-binding protein, sortase A, ESX-1, and serine protase (HtrA), were found in the genome of ZJ0503. Learning more about these factors would strengthen our understanding of the virulence of *N. seriolae*, and analysis of the complete genome sequence of *N. seriolae* strain ZJ0503 will open new avenues for the identification of novel potential vaccine targets.

### Nucleotide sequence accession numbers.

This whole-genome shotgun project has been deposited at DDBJ/EMBL/GenBank the under accession number JNCT00000000. The version described in this paper is the first version and has been assigned accession number JNCT01000000.

## References

[1] Kudo T, Hatai K, Seino A 1988 *Nocardia seriolae* sp. nov. causing nocardiosis of cultured fish. Int J Syst Bacteriol 38:173–178. doi:10.1099/00207713-38-2-173.

[2] Wang GL, Yuan SP, Jin S 2005 Nocardiosis in large yellow croaker, *Larimichthys crocea* (Richardson). J Fish Dis 28:339–345. doi:10.1111/j.1365-2761.2005.00637.x. 15960657

[3] Labrie L, Ng J, Tan Z, Komar C, Ho E, Grisez L 2008 Nocardial infections in fish: an emerging problem in both freshwater and marine aquaculture systems in Asia, p 297–312. *In* Bondad-Reantaso MG, Mohan CV, Crumlish M, Subasinghe RP (ed), Diseases in Asian Aquaculture VI. Fish Health Section, Asian Fisheries Society, Manila.

[4] Besemer J, Lomsadze A, Borodovsky M 2001 GeneMarkS: a self-training method for prediction of gene starts in microbial genomes. Implications for finding sequence motifs in regulatory regions. Nucleic Acids Res 29:2607–2618. doi:10.1093/nar/29.12.2607. 11410670 PMC55746

[5] Smit AFA, Hubley R, Green P 1996 RepeatMasker Open-4.0.5. http://www.repeatmasker.org.

[6] Lagesen K, Hallin P, Rødland EA, Stærfeldt HH, Rognes T, Ussery DW 2007 RNAmmer: consistent and rapid annotation of ribosomal RNA genes. Nucleic Acids Res 35:3100–3108. doi:10.1093/nar/gkm160. 17452365 PMC1888812

[7] Lowe TM, Eddy SR 1997 tRNAscan-SE: a program for improved detection of transfer RNA genes in genomic sequence. Nucleic Acids Res 25:955–964. doi:10.1093/nar/25.5.0955. 9023104 PMC146525

[8] Griffiths-Jones S, Moxon S, Marshall M, Khanna A, Eddy SR, Bateman A 2005 Rfam: annotating non-coding RNAs in complete genomes. Nucleic Acids Res 33:D121–D124. doi:10.1093/nar/gki081. 15608160 PMC540035

[9] Ishikawa J, Yamashita A, Mikami Y, Hoshino Y, Kurita H, Hotta K, Shiba T, Hattori M 2004 The complete genomic sequence of *Nocardia farcinica* IFM 10152. Proc Natl Acad Sci USA 101:14925–14930. doi:10.1073/pnas.0406410101. 15466710 PMC522048

